# Study protocol to investigate the effects of testosterone therapy as an adjunct to exercise rehabilitation in hypogonadal males with chronic heart failure

**DOI:** 10.1186/1471-2261-6-46

**Published:** 2006-11-30

**Authors:** John M Saxton, Irena Zwierska, Atish Mathur, Kevin S Channer

**Affiliations:** 1 Centre for Sport and Exercise Science, Sheffield Hallam University, Sheffield, UK; 2 Cardiology Department, Royal Hallamshire Hospital, Sheffield Teaching Hospitals NHS Trust, Sheffield, UK

## Abstract

**Background:**

Testosterone deficiency is a common occurrence in men with chronic heart failure (CHF) and may underpin features of advanced disease, including reduced skeletal muscle mass and fatigue. It is positively correlated with cardiac output and exercise capacity in patients with CHF, whereas a significant improvement in both these parameters has been observed following testosterone replacement therapy. Testosterone therapy has also been shown to reduce circulating levels of inflammatory markers, (TNF-α, sICAM-1 and sVCAM-1) in patients with established coronary artery disease and testosterone deficiency. This pilot study will assess the feasibility of a combined exercise rehabilitation and adjunctive testosterone therapy intervention for evoking improvements in exercise capacity, circulating inflammatory markers, cardiac and skeletal muscle function, indices of psychological health status and quality of life in hypogonadal males with chronic heart failure.

**Methods/design:**

Following ethical approval, 36 patients will be randomly allocated to one of two groups: testosterone or placebo therapy during exercise rehabilitation. A combined programme of moderate intensity aerobic exercise and resistance (strength) training will be used. The primary outcome measure is exercise capacity, assessed using an incremental shuttle walk test. Secondary outcome measures include measures of peak oxygen uptake, cardiac function, lower-limb skeletal muscle contractile function and oxygenation during exercise, circulating inflammatory markers, psychological health status and quality of life.

**Discussion:**

Exercise rehabilitation can safely increase exercise capacity in stable CHF patients but there is a need for studies which are aimed at evaluating the long-term effects of physical training on functional status, morbidity and mortality. This pilot study will provide valuable preliminary data on the efficacy of testosterone therapy as an adjunct to exercise rehabilitation on a range of functional, physiological and health-related outcomes in this patient population. Preliminary data will be used in the design of a large-scale randomised controlled trial, aimed at informing clinical practice with respect to optimisation of exercise rehabilitation in this patient group.

## Background

Chronic heart failure (CHF) is a common, debilitating condition and is a major public health burden in the Western world. It is a multi-organ disease, involving the musculoskeletal, respiratory and endocrine systems [1]. CHF most frequently results from coronary artery disease or hypertension and patients generally experience a continuing decline in their health, resulting in an increased frequency of hospitalization and premature death. As cardiac transplantation is the only option for long-term survival in patients with CHF, there is a clear requirement for new strategies aimed at altering disease progression, relieving symptoms and prolonging life.

Previous studies have reported testosterone deficiency in men with CHF [2,3], which might underpin features of advanced CHF such as reduced skeletal muscle mass and fatigue [3]. Furthermore, testosterone deficiency is positively correlated with cardiac output [4] and exercise capacity [3] in patients with CHF and a significant improvement in both these parameters has been observed following testosterone replacement therapy [1,3,5]. Although the mechanisms are poorly understood, the improvement in exercise capacity was shown to be positively correlated with the increase in serum testosterone level [3] and was accompanied by a small increase in internal left ventricular length in a recent study [3]. Additional evidence from animal studies suggests that anabolic androgens can attenuate skeletal muscle fatigue in response to exercise [6]. Testosterone replacement therapy has also been shown to reduce circulating levels of inflammatory mediators, such as TNF-α and IL-1β as well as total cholesterol in patients with established coronary artery disease (CAD) and testosterone deficiency [7,8]. Circulating levels of inflammatory mediators are elevated in CHF and may be related to endothelial dysfunction and clinical deterioration in these patients.

Interestingly, chronic administration of low physiologic replacement doses of testosterone can delay the time to ischaemic threshold during treadmill walking in elderly males with established CAD [7]. A rapid mode of action is indicated, as improvements in ischaemic threshold have been observed after as little as one month of intramuscular testosterone replacement compared with placebo [7]. As testosterone is a vasodilator, this could explain its anti-ischemic effects on cardiac function during exercise. However, it is currently unknown whether the vasodilatory effects of testosterone can influence the fatigability of skeletal muscle in a similar fashion.

It is now widely accepted that exercise training can safely increase exercise capacity in stable CHF patients [9]. An improvement in skeletal muscle strength and endurance has been reported after resistance training regimens [10,11]. More recent studies have also reported improvements in dynamic quadriceps and hamstrings strength and endurance following combined aerobic and resistance training programmes [12] and provided evidence that this combined training approach is superior to aerobic training alone for improvement of left ventricular function in patients with CHF [13]. There is also evidence that short-term programmes of exercise training lasting for 12 weeks can reduce the circulating levels of inflammatory mediators such as TNF-α, sICAM-1 and sVCAM-1, which are elevated in CHF [14,15]. However, a need for more clinical trials aimed at evaluating the long-term effects of physical training on functional status, morbidity and mortality in stable CHF patients has recently been highlighted [9]. Considering the low functional capacity of male CHF patients [3], an investigation of strategies that have the potential to augment the response to exercise rehabilitation is warranted in this patient group.

A significant proportion of men with CHF have low testosterone levels. Given the positive effects of testosterone therapy on exercise capacity in CHF[3] and established CAD [7], and considering its positive effects on circulating inflammatory mediators in CAD and testosterone deficiency [7,8], we hypothesize that adjunctive testosterone therapy will augment the positive effects of exercise rehabilitation on these clinical outcomes in hypogonadal males with stable CHF.

## Methods/design

### Primary aims of the study

1. To assess the feasibility of a combined exercise rehabilitation and adjunctive testosterone therapy intervention for evoking improvements in exercise capacity, circulating inflammatory markers, cardiac and skeletal muscle function, indices of psychological health status and quality of life in hypogonadal males with chronic heart failure.

2. To obtain estimates of the variability of the primary outcome measure and an insight into the treatment effect resulting from combined testosterone therapy/exercise rehabilitation intervention versus exercise therapy alone, with the data being used in a subsequent power calculation for a larger-scale randomised controlled trial.

### Patient recruitment

A total of 36 ambulant male patients, over 18 years, with stable CHF (longer than 6 months) and with a blood testosterone level of less than 12 nmol.l^-1 ^and symptoms of hypogonadism will be recruited from clinics at the Royal Hallamshire Hospital, Sheffield. Patients satisfying the inclusion/exclusion criteria will be sent a letter, with an attached patient information sheet. Those patients with a possible interest in study participation will be invited to an initial consultation session. This session will answer any questions which the patient may have, and will familiarise patients with the study protocol and equipment. Before entering the trial, each patient will have a thorough medical examination performed by a Consultant Cardiologist or Research Registrar, during which details of surgical history, co-morbid conditions, risk factors and current medication details will be confirmed. Hormone profiles and drug use will also be recorded at the beginning of the trial. Blood pressure will be taken (manual sphygmomanometer) and a resting 12-lead ECG will be performed with the patient in the supine position. Clinical Trial Authorisation was granted by the Medicines and Healthcare products Regulatory Agency and Ethical approval obtained from the North Sheffield Research Ethics Committee. Informed consent will be obtained from all participants before they enter the study.

### Randomisation

Patients will be randomly allocated to one of the two groups: (i) testosterone replacement therapy during a 12 week programme of exercise rehabilitation, or (ii) 12 week programme of exercise rehabilitation with 'placebo therapy'. Blocked randomisation by a third party, using a pre-determined randomisation schedule, will be used to ensure equal distribution of patients throughout the recruitment process.

### Details of power calculations and sample size

Data on exercise capacity (shuttle-walk performance) for stable CHF patients with testosterone deficiency are unavailable. However, a recent preliminary study reported a statistically significant improvement in shuttle-walk performance in 10 CHF patients following 12 weeks of testosterone therapy [16]. The mean (95% confidence interval) treatment effect was 65 m (12.6 – 117.4 m). In the proposed study, a main objective of the planned analysis is to obtain estimates of the variability of the primary outcome measure and an insight into the treatment effect resulting from combined testosterone therapy/exercise rehabilitation, with the data being used in a subsequent power calculation for a larger-scale randomised controlled trial. Allowing for a possible 30% drop-out over the course of the intervention, recruitment of 18 patients for each group will enable this sample size calculation to be performed.

### Patient inclusion criteria

a. Ambulant male patients with symptoms of hypogonadism

b. Blood testosterone level of less than 12 nmol.l^-1^

c. Clinically stable CHF (longer than 6 months)

d. Evidence of impairment of left ventricular systolic function (defined by echocardiography as ejection fraction less than 35%)

e. Reduced exercise tolerance (limited by fatigue or breathlessness of cardiac origin)

f. Over 18 years of age

### Patient exclusion criteria

a. Unstable angina

b. Recent acute myocardial infarction

c. Decompensated heart failure

d. Haemodynamically significant valvular heart disease

e. Uncontrolled hypertension

f. Renal insufficiency (serum creatinine >2.5 mg.dl^-1^)

g. Urologic disorders

h. Any orthopaedic or neurologic illness limiting the ability to exercise

i. A prostate specific antigen (PSA) level above the age adjusted normal range

j. Patients taking certain concurrent medications might also be excluded

### Supervised exercise

Cycle ergometry will constitute the main component of the aerobic exercise training regimen. Exercise intensity will be set at 50% of the maximum short-term exercise performance, determined by a steep ramp test every 4 weeks (3 minutes of unloaded pedalling, then work rate increments of 12.5 W every 10 seconds at a crank rate of 60 rpm) [12]. An interval training regimen will be used, incorporating 30 seconds of exercise with one minute interpolated rest periods for a total exercise time of 15 minutes (i.e. 10 repeats) [9]. Each aerobic exercise session will be followed by two sets of low to moderate intensity resistance exercises for five main muscle groups: quadriceps, hamstrings, pectorals, latissimus dorsi, deltoids. The supervised exercise sessions will last for approximately 45–50 minutes in total and patients will be required to attend 3 sessions per week. The venue for supervised exercise will be the Exercise Science Laboratory at Sheffield Hallam University, which has showering facilities and is on a major bus route close to the City centre.

### Testosterone replacement therapy

Patients will be administered testosterone therapy or placebo in an identical fashion and will be blinded to which treatment they are receiving. Testosterone (Sustanon 100, 100 mg testosterone/ml; Organon Laboratories Ltd, Cambridge, UK) or placebo (1 ml of 0.9% normal saline) in an identical syringe will be given by deep intramuscular injection to the buttock every two weeks by a member of the research staff not involved in the delivery of supervised exercise or assessment of outcomes. Patients will be blinded to the identity of the injection and the drug will be drawn up away from the patient.

### Outcome measures

Unless otherwise stated, outcome measures will be assessed at baseline and 12 weeks in all patients. Members of the research team responsible for assessment of the primary and secondary outcomes and for delivering the supervised exercise training will be blinded to group allocation.

### Primary outcome measure

#### Exercise capacity

The primary outcome measure is exercise capacity, assessed using the incremental shuttle walk test (ISWT), as described in our recently published work [3]. Two tests will be performed prior to starting treatment, one at the screening visit and the second before randomisation, which will be used as the baseline result in subsequent analysis. The ISWT is a symptom-limited exercise test with a progressive increase in workload designed to allow subjects to achieve maximum effort tolerance. Subjects walk back and forth along a horizontal 10 metre course, marked out by two cones and must complete the shuttle before a pre-recorded signal from a cassette player, which shortens incrementally after each shuttle. The end-point (distance walked in metres) is reached when the subject fails to complete the shuttle before the signal. The ISWT has been evaluated in patients with chronic heart failure, as an alternative to cardiopulmonary exercise testing and the traditional 6-minute walk test. It is highly reproducible, preferred by patients, correlates strongly with peak VO_2 _(r = 0.84, p < 0.0001) and, after 17 months follow up, was shown to predict event free survival.

### Other outcome measures

#### Cardiopulmonary function, skeletal muscle and cardiac function

Peak oxygen consumption (V˙
 MathType@MTEF@5@5@+=feaafiart1ev1aaatCvAUfKttLearuWrP9MDH5MBPbIqV92AaeXatLxBI9gBaebbnrfifHhDYfgasaacH8akY=wiFfYdH8Gipec8Eeeu0xXdbba9frFj0=OqFfea0dXdd9vqai=hGuQ8kuc9pgc9s8qqaq=dirpe0xb9q8qiLsFr0=vr0=vr0dc8meaabaqaciaacaGaaeqabaqabeGadaaakeaacuqGwbGvgaGaaaaa@2DE8@ O_2_) will be assessed using incremental cycle ergometry (Excalibur, Lode, Groningen, The Netherlands). The test will begin at a power output of 25 W and increase by 25 W every 3 minutes to maximal exercise tolerance. The test will be terminated when the patient becomes restricted by clinical symptoms or the crank rate of 60 rpm cannot be maintained. Blood pressure, heart rate, perceived exertion (Borg scale range 6–20) and 12-lead ECG recordings (Marquette CaSE 15, Wisconsin, U.S.A.) will be made after 10 minutes of supine rest and within the last 30 seconds of each work-rate increment. Pulmonary gas exchange variables will be measured breath-by-breath (CaSE EX670 PulmoLab, Kent, U.K.) for assessment of peak V˙
 MathType@MTEF@5@5@+=feaafiart1ev1aaatCvAUfKttLearuWrP9MDH5MBPbIqV92AaeXatLxBI9gBaebbnrfifHhDYfgasaacH8akY=wiFfYdH8Gipec8Eeeu0xXdbba9frFj0=OqFfea0dXdd9vqai=hGuQ8kuc9pgc9s8qqaq=dirpe0xb9q8qiLsFr0=vr0=vr0dc8meaabaqaciaacaGaaeqabaqabeGadaaakeaacuqGwbGvgaGaaaaa@2DE8@ O_2 _and ventilatory threshold (V_T_). V_T _is used to detect the beginning of excess CO_2 _production resulting from the buffering of H^+ ^arising from lactic acid production. Local muscle haemoglobin saturation will be monitored at rest, during exercise and recovery using near infrared spectroscopy (NIRS; NIRO 300, Hamamatsu, Welwyn Garden, UK). This is a non-invasive technique, in which a light emitting/detecting probe is placed on the skin over the vastus lateralis muscle. The NIRS signal is mainly derived from the haemoglobin in the microvasculature (precapillary, capillary, postcapillary) of the sampled tissue and reflects local tissue oxygenation.

Dynamic muscular strength and endurance of the dominant leg quadriceps and hamstrings will be measured using an isokinetic dynamometer (Biodex System 3 dynamometer, Biodex Medical, NY, USA). The test protocol will comprise a 5-minute warm-up on a cycle ergometer, 3 repetitions at an angular velocity of 1.0 rad.s^-1^, 5 repetitions at 3.0 rad.s^-1 ^and 20 repetitions at 5.3 rad.s^-1^, followed by a cool-down on a cycle ergometer. A 2-minute rest period will be applied between each set of repetitions and the main outcome measures will be peak torque and total amount of muscular work (endurance) performed at each angular velocity. This protocol was recently shown to be safe for measuring skeletal muscle strength and endurance in CHF patients [12]. This protocol will take approximately 15 – 20 minutes to complete. Isometric strength of the forearm muscles will also be assessed using a hand-grip dynamometer.

Morphological changes in cardiac function, with special emphasis on long axis function, will be assessed using 2D Doppler echocardiographic scanning techniques [17].

### Circulating adhesion molecules and inflammatory mediators

Venous blood samples, for analysis of total testosterone, sex hormone binding globulin, soluble adhesion molecules (sICAM-1, sVCAM-1) and the systemic inflammatory mediators (TNF-α and CRP) by commercially-available enzyme immunoassay kits, will be collected after an overnight fast between 0800 and 0930. Full blood count, glucose concentration, brain natiuretic peptide, and lipid profiles will also be measured at these time points using hospital assays. All blood samples will be collected at least 72 hours after the last supervised exercise bout. Bioavailable testosterone will be calculated by a modification of the method described by Tremblay and Dube [18]. Additional blood samples will be collected during weeks 4 and 8 of the intervention, for analysis of testosterone status and PSA levels.

### Psychological health status and quality of life

Both CHF and androgen deficiency are characterised by low mood and depression, which is improved by testosterone replacement therapy in testosterone deficient subjects. Thus, at baseline and 12 weeks, patients will be asked to complete the Minnesota living with heart failure questionnaire, the SF-36 *v2 *Health Survey, the Beck Depression Inventory and the Androgen Deficiency in the Adult Male Screening Questionnaire. Physical activity behaviour in everyday life will be measured at baseline, 4, 8, and 12 weeks by the Community Healthy Activities Model Program for Seniors Questionnaire (CHAMPS) Physical Activity Questionnaire for older adults. After the intervention, patients will also be interviewed about their subjective experiences and views on which aspects of the intervention worked and which aspects could be improved.

### Analysis of results

As previously stated, a main objective of the planned analysis is to obtain estimates of the variability of the primary outcome measure and an insight into the treatment effect resulting from combined testosterone therapy/exercise rehabilitation, with the data being used in a subsequent power calculation for a larger-scale randomised controlled trial. Primary and secondary outcomes will also be compared at each follow-up point using analysis of covariance procedures, with baseline values being used as the covariate. In addition, bivariate relationships between physical activity dose and other outcome variables will be analysed using appropriate correlation techniques. Statistical significance will be set at *p *< 0.05. All data will be analysed using the SPSS statistical package (SPSS UK Ltd, Woking, U.K.).

## Discussion

The rapidly growing ageing population in Western societies will inevitably be receiving pharmacological therapy for chronic heart conditions and vascular diseases. However, such individuals can still benefit greatly from engaging in a physically active lifestyle. This highlights the need for more studies into the benefits and risks of combined programmes of exercise rehabilitation and pharmacological therapies to build a strong foundation for evidence-based therapy.

Testosterone deficiency is common in men with CHF and could influence the adverse effects of the condition on skeletal muscle size and fatigability. We have recently completed a proof-of-concept clinical study showing that testosterone replacement therapy improves effort tolerance in men with CHF [3]. However, the full potential of this therapeutic approach for improving physical function and quality of life in patients with CHF is not yet known. This pilot study will provide valuable preliminary data on the efficacy of testosterone therapy as an adjunct to exercise rehabilitation on a range of functional, physiological and health-related outcomes, with the preliminary data being used in the design of a larger-scale trial. The proposed investigation is very timely and we expect the results to ultimately inform clinical practice with respect to optimisation of exercise rehabilitation in this patient group.

## Competing interests

The author(s) declare that they have no competing interests.

## Authors' contributions

JS and KC conceived and drafted the research proposal and sought funding for the study. JS is responsible for overall project management and KC is responsible for overseeing all clinical issues. IZ and AM contributed intellectual input into the research protocol and will have a major role in the recruitment of volunteers, assessment of outcomes and implementation of the exercise training programme. All authors read and approved the final manuscript.

**Figure 1:**
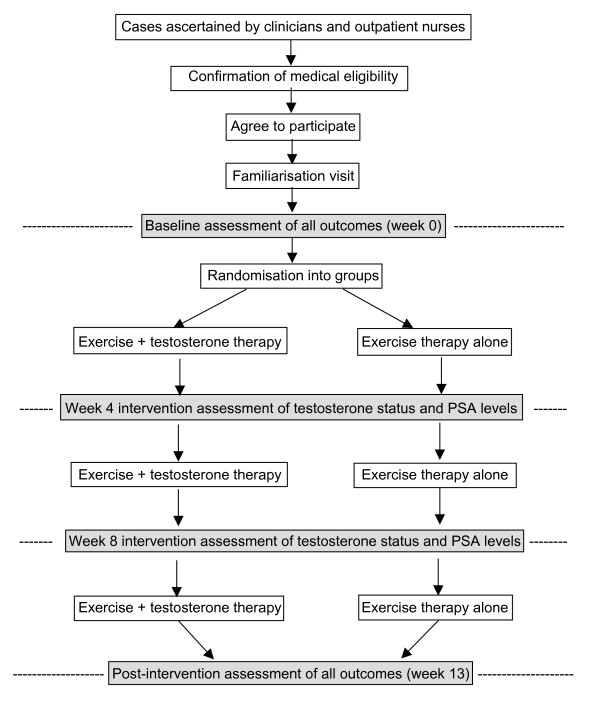
Flow chart showing the study design.

## Pre-publication history

The pre-publication history for this paper can be accessed here:


